# Knowledge-Guided Robust MRI Brain Extraction for Diverse Large-Scale Neuroimaging Studies on Humans and Non-Human Primates

**DOI:** 10.1371/journal.pone.0077810

**Published:** 2014-01-29

**Authors:** Yaping Wang, Jingxin Nie, Pew-Thian Yap, Gang Li, Feng Shi, Xiujuan Geng, Lei Guo, Dinggang Shen, for the Alzheimer's Disease Neuroimaging Initiative

**Affiliations:** 1 School of Automation, Northwestern Polytechnical University, Xi'an, Shaanxi Province, China; 2 Department of Radiology and BRIC, University of North Carolina at Chapel Hill, North Carolina, United States of America; 3 Neuroimaging Research Branch, National Institute on Drug Abuse, Baltimore, Maryland, United States of America; 4 Department of Brain and Cognitive Engineering, Korea University, Seoul, Korea; Cuban Neuroscience Center, Cuba

## Abstract

Accurate and robust brain extraction is a critical step in most neuroimaging analysis pipelines. In particular, for the large-scale multi-site neuroimaging studies involving a significant number of subjects with diverse age and diagnostic groups, accurate and robust extraction of the brain automatically and consistently is highly desirable. In this paper, we introduce population-specific probability maps to guide the brain extraction of diverse subject groups, including both healthy and diseased adult human populations, both developing and aging human populations, as well as non-human primates. Specifically, the proposed method combines an atlas-based approach, for coarse skull-stripping, with a deformable-surface-based approach that is guided by local intensity information and population-specific prior information learned from a set of real brain images for more localized refinement. Comprehensive quantitative evaluations were performed on the diverse large-scale populations of ADNI dataset with over 800 subjects (55∼90 years of age, multi-site, various diagnosis groups), OASIS dataset with over 400 subjects (18∼96 years of age, wide age range, various diagnosis groups), and NIH pediatrics dataset with 150 subjects (5∼18 years of age, multi-site, wide age range as a complementary age group to the adult dataset). The results demonstrate that our method consistently yields the best overall results across almost the entire human life span, with only a single set of parameters. To demonstrate its capability to work on non-human primates, the proposed method is further evaluated using a rhesus macaque dataset with 20 subjects. Quantitative comparisons with popularly used state-of-the-art methods, including BET, Two-pass BET, BET-B, BSE, HWA, ROBEX and AFNI, demonstrate that the proposed method performs favorably with superior performance on all testing datasets, indicating its robustness and effectiveness.

## Introduction

Brain extraction (also known as skull stripping), is an important preprocessing procedure in brain magnetic resonance (MR) image analysis. It aims to remove non-brain tissues, such as skull, dura and eyes, and retain the brain tissues, typically in a T1-weighted brain MRI scan. Extraction of the brain in an MRI scan is a difficult problem due to the complex nature of the brain image. Especially, when applied to diverse large-scale datasets with varying scanning parameters, different age and diagnostic groups, many existing methods may only work well on certain datasets with certain parameter settings and thus a tremendous amount of human intervention is needed for parameter tuning across datasets. Moreover, these ‘optimized’ parameters do not guarantee satisfactory results. As the first component in most neuroimaging processing pipelines, the accuracy of brain extraction is crucial to many subsequent processing steps such as brain tissue segmentation, brain image registration, cortical surface reconstruction, brain morphometry, and disease diagnosis [1], [2], [3], [4], [5]. For example, incorrectly removing brain tissues could result in under-estimation of the cortical thickness, whereas, incorrectly retaining non-brain tissues might lead to over-estimation of the cortical thickness [1], [2], [3]. It should be noted that over skull stripping cannot be rectified in subsequent processing steps. Thus an accurate and robust approach for the extraction of the brain automatically and consistently is highly desirable especially for diverse large-scale multi-site studies, such as the Alzheimer's Disease Neuroimaging Initiative (ADNI) dataset [6], Open Access Series of Imaging Studies (OASIS) dataset [7], and NIH Pediatric Database (NIHPD) [8], which could greatly reduce the need for intensive human intervention that is quite time-consuming and may cause bias or inconsistency.

Numerous methods [9], [10], [11], [12], [13], [14], [15], [16], [17], [18], [19], [20], [21], [22], [23], [24], [25], [26], [27], [28], [29], [30], [31], [32], [33] have been proposed for brain extraction in last several decades. Bomans et al. (1990) [10] presented a 3D segmentation and reconstruction method for the anatomical surface of MRI data, in which a 3D extension of the Marr-Hildreth operator is used to detect the brain surface, and morphological filters are also applied to improve the surface definition. Other morphology-based method are also proposed [9], [11], [12], [13], [14], [15], [16] following this direction. Kapur et al. (1996) [13] presented a method that combines three existing techniques from the computer vision literature: expectation maximization segmentation, binary mathematical morphology, and active contour models. Atkins and Mackiewich (1998) [11] integrate the thresholding, morphological operations and “snakes” techniques in a multistage process involving: the removal of the background noise leaving a head mask; finding a rough outline of the brain; and the refinement of the brain outline to obtain a final mask. Lemieux et al. (1999) [14] adopt a more sophisticated version, with each step carefully tuned to overcome a specific problem. For example, they deal with thin strands by thresholding at the gray matter level, followed by morphological opening and connected component analysis. A surface-based method is also introduced [22], which involves deforming a tessellated ellipsoidal template into the shape of the inner surface of the skull, followed by iterative deformation driven by intensity-based force and curvature-based force. Hahn and Peitgen (2000) [19] proposed a 3D watershed transform algorithm, which combines with pre-flooding to avoid over-segmentation. A popular method called Brain Surface Extractor (BSE) is proposed by Shattuck et al. (2001) [18], introducing anisotropic diffusion filtering as a denoising step prior to edge detection, and mathematical morphological operations, for increased robustness. Another popular method, called the Brain Extraction Tool (BET) [23], uses a deformable model that evolves a surface to fit the brain boundary by application of a set of locally adaptive forces, accounting for surface smoothness and voxel intensity changes in the surface vicinity. Although morphology-based methods can be effective, they generally require some degree of user interaction and are sensitive to scanning parameters as well as intensity inhomogeneities. For surface-based methods, they are generally more robust and less sensitive to image artifacts, and require less human interaction [22], [23].

More recently, Rehm et al. (2004) [20] proposed a method, which incorporates atlas-based extraction via nonlinear warping, intensity-threshold masking with connectivity constraints, and edge-based masking with morphological operations. A hybrid approach, called Hybrid Watershed Algorithm (HWA) [25] is proposed, combining the watershed algorithm [19] with a deformable-surface model using statistics of the surface curvature and the distance of the surface to the center of gravity (COG) to detect and correct inaccuracies in brain extraction. Meta methods have been introduced to combine skull-stripping results from different approaches to reduce susceptibility to bias or errors. For instance, Rex et al. (2004) [28] combine results from brain extraction algorithms such as BSE, BET, AFNI's 3dIntracranial, and HWA for better skull-stripping outcome. Chiverton et al. (2007) [29] describe a novel automatic statistical morphology skull stripping method, utilizing statistical techniques including fitting of probabilistic functions and thresholding. Sadananthan et al. (2010) [21] introduced a graph cuts based method, which utilizes intensity thresholding followed by removal of narrow connection using a graph theoretic image segmentation technique. Leung et al. [27] utilize a multi-atlas based label propagation and label fusion method to extract the brain. A brain extraction algorithm is proposed in [26], combining elastic registration, tissue segmentation, and morphological techniques by a watershed-based framework. Iglesias et al. (2011) [33] introduced a learning-based brain extraction system called ROBEX, which combines a discriminative random forest classifier and a generative point distribution model. However, most of existing techniques do not give consistently satisfactory results over a wide range of scan types and neuroanatomies without some forms of manual intervention, due to the presence of imaging artifacts, anatomical variability, and contrast variation. Especially for methods relying on the image intensity, their performance may be influenced by numerous factors including signal inhomogeneities, stability of system electronics, and the extent of neurodegeneration [2]. Suboptimal outcomes in these circumstances often require further manual parameter adjustment for refining brain extraction results across different datasets.

In this paper, we propose a population-specific prior-knowledge guided deformable-surface-based framework for accurate and robust brain extraction with applications to diverse populations. Due to the complexity of the brain, methods that rely on intensity information alone are relatively susceptible to local minima. For more robust surface-deformation, population-specific probability maps are employed to guide the skull-stripping process using topological constraints and realistic shapes. This is complementary to the local intensity information, which helps accurately localize the brain boundary in different individuals, compensating for inter-subject variations. The combination of all these pieces of information compensates for the weaknesses of the individual components and hence can help achieve better results. To achieve this, we first build a population-specific brain probability map, which encapsulates prior information gathered from a population of real brain MR images by warping the manual extracted brains of individual images to the atlas space. For good spatial initialization, affine (FLIRT [34]) and nonlinear registration (Demons [35]) are utilized to warp the atlas to the subject image. The brain mask obtained from the binarized brain probability map accompanying the atlas is warped to the subject image for initial skull stripping. The brain probability map is further employed to guide surface evolution, in combination with surface geometry and intensity information, for refinement of the skull-stripping result. In summary, our method involves an initial brain extraction by co-registration of an atlas, followed by population-specific prior-information guided surface deformation for localized skull-stripping refinement.

A preliminary version of this work was presented as a conference article in [36]. The current work is an extension of the previous work. All results are new and were generated using this new extended method. First, the proposed method is improved and optimized by introducing population-specific probability maps so that the method is applicable to diverse groups such as healthy and diseased, developing and aging human populations, as well as non-human primates. Specifically, for constructing the probability map, a rescaling step based on the distance transform map is further introduced to account for the inter-subject variation and potential estimation inaccuracy due to the insufficient number of training images. Second, the proposed method was extensively evaluated on four diverse datasets, including ADNI, OASIS, NIH pediatric dataset, and a rhesus macaque dataset. These datasets cover almost the whole human life span and also nonhuman primates. The consistently good performance of the proposed method among the diverse groups of images demonstrates its robustness and wide applicability. Third, besides Dice coefficient, additional different metrics were employed for multi-faceted performance evaluation of the proposed method, including false positive and false negative spatial probability maps, mean symmetric and maximal surface-to-surface distances. Extensive evaluation of the parameter sensitivity is also included. Fourth, comparisons were performed with other 7 popularly used state-of-the-art methods, including BET, Two-pass BET, BET-B, BSE, HWA, ROBEX, and AFNI. Experimental results indicate that the proposed method significantly outperforms all compared methods, with one fixed set of parameters for each dataset. On the contrary, for all other methods, optimized parameters determined by grid search were employed for each image. Details are provided in the following sections.

## Subjects and Data Acquisition

To extensively evaluate the proposed method, four diverse and large-scale datasets: ADNI, OASIS, NIH pediatrics, and rhesus macaque, were used for evaluation. **Table 1** shows the demographics and acquisition protocols for all four datasets. Here we mainly focus on T1-weighted MR images, since T1-weighted imaging is the most frequently used structural MRI modality and is often used as the reference for other modalities in neuroimaging studies [33].

**Table 1:** Demographics and acquisition protocols of all four datasets used in this study. Note that ND-Y, ND-M, and ND-E denote young adults, middle-aged adults, and elderly adults in the non-demented group of the OASIS dataset, respectively.

Dataset	ADNI				OASIS					NIHPD	Rhesus Macaque
	HC	MCI	AD	All	ND-Y	ND-M	ND-E	Demented	All		
**No. of Subjects**	230	406	199	835	154	66	96	100	416	150	20
**Age range (years)**	55∼90				18∼39	40∼60	61∼94	62∼96	18∼96	5∼18	24∼30 (months)
**Scanner**	GE/Philips/Siemens				Siemens					GE/Siemens	Siemens
**MR strength (T)**	1.5				1.5					1.5	3.0
**Modality**	T1				T1					T1	T1
**Sequence**	MP-RAGE				MP-RAGE					SPGR	MP-RAGE

### ADNI

The Alzheimer's Disease Neuroimaging Initiative (ADNI, www.adni-info.org) includes more than 800 participants with an age range of 55–90, recruited from over 50 sites across the U.S. and Canada. The primary goal of ADNI is to determine whether neuroimaging assessments can accurately measure the progression of mild cognitive impairment (MCI) and early Alzheimer's Disease (AD). T1-weighted MRI scans of 835 participants were downloaded from the ADNI public website (http://www.loni.ucla.edu/ADNI/). The downloaded data initially included 230 healthy controls (HC), 406 patients with MCI, 199 patients with AD. The baseline T1-weighted volumetric scans were used for this study. Data was acquired from 1.5T GE, Philips, and Siemens MRI scanners using a sagittal 3D magnetization prepared rapid acquisition gradient echo (MP-RAGE) sequence [37]. Representative imaging parameters were TR = 2300 ms, TI = 1000 ms, TE = 3.5 ms, flip angle = 8°, field of view (FoV) = 240×240 mm, and 160 sagittal slices with a 192×192 matrix yielding a voxel resolution of 1.25×1.25×1.2 mm^3^, or 160–180 sagittal slices with a 256×256 matrix yielding a voxel resolution of 0.94×0.94×1.2 mm^3^ (scan parameters vary between sites, scanner platforms, and software versions). Further details regarding the ADNI MR imaging protocol can be found in Jack et al. (2008) [37]. For consistency, all images were resampled to dimensions 256×256×256 and resolution 1×1×1 mm^3^. Nonparametric nonuniform intensity normalization (N3) [38] was performed for correction of intensity inhomogeneity. Manual skull stripping of all the images was performed semi-automatically by an expert. Specifically, for each image, an initial coarse brain mask was generated using publicly available software packages: BET [23] and BSE [18]. The mask was then manually edited with ITK-SNAP [39] to ensure accurate skull removal. The expert checked the whole brain slice-by-slice to rectify incorrectly segmented regions, recovering over-segmented brain tissues and removing non-brain tissues (e.g., dura). It took the expert around 6 months to completely process all images. Since the initial masks were very coarse and were significantly refined during manual editing, the bias introduced by the software packages is minimal.

### OASIS

The Open Access Series of Imaging Studies (OASIS, http://www.oasis-brains.org/) is a project aimed at making MRI data sets of the brain freely available to the scientific community and facilitating future discoveries in basic and clinical neuroscience. The cross-sectional MRI dataset of the OASIS project, including young, middle-aged, non-demented and demented older adults, is used here. This set consists of a cross-sectional collection of 416 subjects aged 18 to 96 years. 100 of the subjects over the age of 60 were clinically diagnosed to show very mild to moderate symptoms of Alzheimer's disease (AD). The T1-weighted scans were acquired on a 1.5T Siemens Vision scanner with a MP-RAGE sequence, TR/TE/TI/TD = 9.7 ms/4.0 ms/20 ms/200 ms, flip angle = 10°, 128 sagittal 1.25 mm-thick-slices and a 256×256 matrix yielding a voxel resolution of 1.0×1.0×1.25 mm^3^ [7]. All images were resampled into 1 mm isotropic images with dimensions 176×208×176. Intensity inhomogeneity correction was performed for all images [7] using a parametric bias field correction method described in [40]. The brain masks from OASIS were constructed with an atlas-registration-based method and were reviewed by human experts to ensure accuracy [7], [33]. We further divided the non-demented group into three age groups: young adults (154 subjects; age range: 18∼39 years), middle-aged adults (66 subjects; age range: 40∼60 years), elderly adults (96 subjects; age range: 61–94 years). 100 subjects in the demented group (age range: 62∼96 years) were also investigated. This dataset consists of scans from a highly diverse population with a wide age range as well as different diagnosis groups, thus it is valuable to evaluate our algorithm [33].

### NIH Pediatric Database

NIH Pediatric Database (NIHPD) for the study of normal brain development [8] is a multi-center imaging data collected at six Pediatric Study Centers, using 1.5T GE or Siemens scanner. T1-weighted Spoiled Gradient Recalled (SPGR) echo sequence was performed on each participant, with 1 mm isotropic data acquired sagittally from the entire head. Slice thickness was set to ∼1.5 mm for GE scanners due to their limit of 124 slices. The dataset is publicly available at www.pediatricmri.nih.gov. 150 subjects were randomly selected from this study with ages ranging from 5 to 18 years old. All the images were processed using an in-house automated image processing pipeline by Montreal Neurological Institute of McGill University [8]. Correction for image intensity non-uniformity was performed firstly. The brain masks were identified in native space, followed by manually checking and editing for quality control. For consistency, all images were resampled to the dimension of 256×256×256 and resolution of 1×1×1 mm^3^.

### Rhesus Macaque

Twenty rhesus macaques aged 24–30 months were included in this study. All monkeys were healthy with no known pathological conditions. These monkeys were born and housed at the National Institutes of Health Animal Center in Poolesville, Maryland. They were raised together in a large social group including adult, juvenile, and infant monkeys, which allowed visual, auditory, somatosensory, and social interactions with familiar animals. Animals lived in indoor-outdoor pens composed of welded galvanized steel mesh connected by guillotine doors [41]. The indoor-outdoor cages were equipped with a variety of climbing and perch substrates and toys. Monkeys were fed Purina™ High Protein Monkey Chow and received water ad libitum. Supplemental fresh fruits, vegetables, sunflower seeds and Primatreats™ were provided twice daily. The health of each animal was monitored daily by the researchers and the animal care staff, and was checked twice daily by the veterinarians. Protocols were approved by the Institutional Animal Care and Use Committee (IACUC) of the National Institute on Alcohol Abuse and Alcoholism, the National Institute on Drug Abuse, the National Institute of Child Health and Human Development, and National Institutes of Health and Human Services.

T1-weighted MR brain images were acquired using a 3.0 T scanner (Allegra; Siemens Medical Solutions, Inc, Malvern, Pennsylvania), with the following parameters: repetition time/echo time/inversion time, 2500/3.49/1000 ms; 1 slab of 224 sections, 0.6 mm section thickness, 0.3 mm spacing, 8° flip angle, 256×256 pixel acquisition matrix, with the resolution 0.39×0.39×0.6 mm^3^. The images were resampled to the resolution of 0.39×0.39×0.39 mm^3^, and all images were oriented in a standardized oblique plane to eliminate any bias in section angle. In the standardized orientation, the trans-axial plane was parallel to the anteroposterior commissures line and perpendicular to the inter-hemispheric fissure. A threshold-based region-growing algorithm was used to outline the brain in each axial section, following with manual editing for accurately excluding the skull and dura. The above processing was performed with Analyze 7.5 (Biomedical Imaging Resource, Mayo Foundation, Rochester, Minnesota).

## Methods

**Figure 1:**
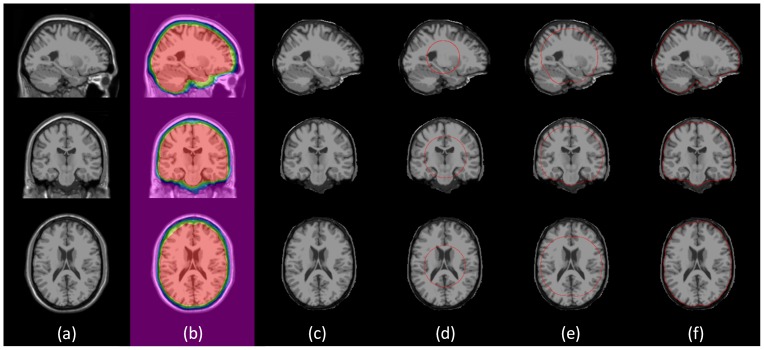
Illustration of the proposed method. From left to right: (a) the original with-skull image; (b) the original with-skull image, with the probability map overlaid; (c) the initial brain extraction result; (d) the initial surface shown in red; (e) the intermediate surface generated as deformation progresses; (f) the final surface.

**Fig. 1** illustrates the different stages of the proposed brain extraction method. First, a brain probability map (**Fig. 1**b and **Fig. 4**), which is constructed by warping a set of real brain MR images along with their manually delineated brain masks to the template space, is used to mask the original with-skull image (**Fig. 1**a) for approximate skull-stripping (**Fig. 1**c) and surface initialization. This is followed by a refinement with a deformable surface, guided by the brain probability map. The initial surface (**Fig. 1**d) is first constructed according to the radius and center of gravity (COG) estimated based on the approximately skull-stripped image. Then the surface evolves toward the brain boundary gradually driven by the intensity-based force, under the guidance of the probability map (**Fig. 1**e). Finally the brain boundary is located and the brain extraction result is obtained (**Fig. 1**f). It should be noted that the terms “atlas” and “template” are used interchangeably in this paper.

### 3.1 Initialization and Parameter Estimation

Given a subject image, a template image is first registered (affine registration by FLIRT followed by nonlinear registration by Demons) to the subject image and then the brain probability map accompanying the template (binarized with the probability greater than 0) is used to mask the target image for approximate brain extraction, to facilitate more accurate parameter estimation and better positioning of the initial deformable surface. Specifically, for the human datasets, the ICBM (International Consortium for Brain Mapping) high-resolution single subject template (with skull) [42] is used. For the non-human primate dataset, a representative subject is selected as the template. The details of constructing the brain probability map are described in the **Section 3.2.2**. After obtaining the approximately skull-stripping result, it is further refined in the following deformable-surface-based step.

The approximately skull-stripped brain images, where most of the skull and the scalp are removed, are used to estimate a set of parameters which will be used in the following deformable-surface-based step, such as image center, intensity minimum 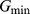, the intensity threshold 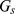 that separates brain and non-brain tissues, and the median intensity 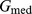. Note that BET [23] estimates these parameters from the raw brain image and is hence more susceptible to distraction caused by non-brain tissues such as the neck. In contrast, these parameters are estimated more accurately in our method with the approximately skull-stripped image, which will benefit the initialization of the deformable surface to avoid local minima and suboptimal solutions.

Inspired by the work of Smith [23], the image intensity minimum 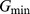 and maximum 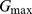 are first estimated from the 2^nd^ and 98^th^ percentiles of the image intensity distribution. 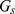, a threshold for roughly separating the brain matter and background, is then defined as 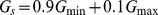. All voxels with intensities between 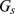 and 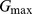 are regarded as brain/head voxels and are hence used as the mass to weight the positions of the voxels for the calculation of the center of gravity (COG). By regarding these voxels as forming a spherical volume, we can estimate the radius of the brain, which is used to initialize the brain surface model. The median intensity 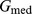 of the brain is calculated from all voxels within the sphere.

With above estimated parameters, the brain surface is modeled by a mesh tessellated using connected triangles [23]. Specifically, a tessellated sphere for the initial model is generated by starting with an icosahedron and further iteratively subdividing each triangle into four smaller triangles. The distance from the center of the sphere to each vertex is adjusted to make the surface as spherical as possible. The initial surface is located at the half of the radius from the COG estimated, with a total of 2562 vertices and 5120 triangles.

### 3.2 Brain Extraction based on Deformable Surface

From the initial position, the surface grows gradually to the target position for one vertex at a time, driven by the following forces. Firstly the intensity-based force obtained from the image intensity information in the surface vicinity is the main force driving surface evolution; secondly the population-specific probability-map-guided force obtained from the warped brain probability map is used to guide the surface evolution; thirdly the smoothness-constrained force is also appended for uniform within-surface vertex spacing and surface smoothness. Each force will be detailed in the following sections one by one.

### 3.2.1 Intensity-based Force

**Figure 2:**
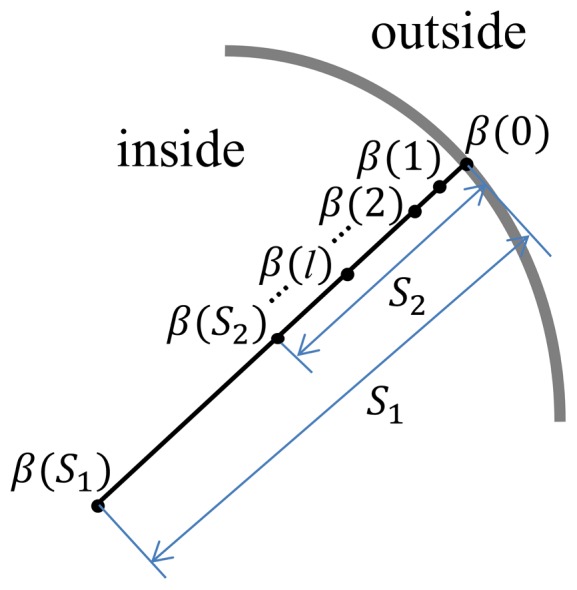
Searching for locally minimal and maximal intensities.


The intensity-based force acts along the local surface normal. It accounts for voxel intensity changes in the surface vicinity to force the surface model to move towards the real brain surface. Following Dale and Smith's work [22], [23], the local minimum intensity 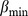 is defined as 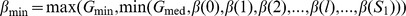, and the local maximum intensity 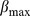 is computed as 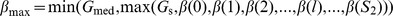. This is achieved by searching along a line that starts from the current vertex and points inwards to the brain along the normal direction as illustrated in **Fig. 2**. Here 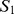 and 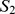 represent for the spatial search ranges pertaining to the minimum and maximum intensities respectively. And 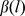 is the intensity of the voxel on the line with 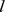 millimeters (mm) away from the current vertex. The distance searched for 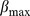 should be long enough to reach the deep sulci and the white matter; on the other hand, the distance searched for 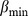 should also be long enough so that the sampling line reaches the CSF when the evolving contour passes the brain surface [24]. Typically, 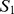 is set to 20 mm and 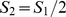 (this ratio is empirically optimized in BET [23]). As described in **Section 3.1**, 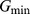, 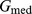, and 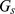 are estimated according to the intensity distribution of the initial skull-stripped brain image. Note that 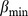 is limited between 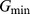 and 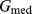; and 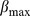 is limited between 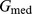 and 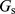. This is to help avoid outlier voxels with intensities that are too dark or too bright.

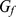
, the locally estimated upper threshold of CSF, is a fraction that lies on the way between 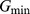 and the local maximum intensity 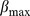. It is defined as:
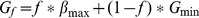
(1)where the parameter *f* is called the *fractional intensity threshold* and falls between the range of 0 and 1. This threshold is used to distinguish between brain and non-brain tissues.

Thus the intensity-based force derived from the image intensity information in the surface vicinity is defined as:
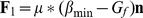
(2)Here **n** is the outward-oriented surface normal at the current vertex, and 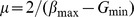 is the normalization term that restricts 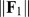 in a certain range. The direction of surface evolution is dependent on 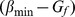. When 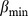 is larger than *G_f_*, indicating that the current vertex is still within the brain, **F**_1_ will attempt to drive the current vertex to move outwards the true brain boundary. When 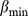 is smaller than *G_f_*, indicating that the current vertex has passed the CSF, **F**_1_ will act in the opposite direction, forcing the current vertex to move inwards.

### 3.2.2 Probability Map Guided Force

First, we introduce **the construction of the population-specific brain probability map**. To better account for the intrinsic characteristics of different diagnostic or age groups, we construct the probability map for each specific group separately. For each specific group, the template image is aligned onto a set of training images (with skulls) first linearly (FLIRT, DOF = 12) [34] and then non-linearly (Demons) [35], respectively. Then the manually delineated brain masks of the training images are warped to the template space using the corresponding inverse transformations. The brain probability map, which indicates the likelihood of each voxel of belonging to a part of the brain, is constructed from the warped manually skull-stripped images (training images). It is worth noting that Demons registration method works especially well in the image regions with clear intensity changes. In a T1-weighted MR image, the brain boundary appears as a surface with low cerebrospinal fluid (CSF) level intensity, compared with the gray matter and the skull, thus providing a good contrast for alignment.

From the aligned brain masks, we evaluate the probability of each voxel belonging to the brain by computing the fraction of brain masks that consider this voxel as a part of the brain. For each voxel 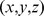, its probability is calculated as:
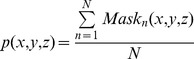
(3)where *N* is the total number of warped brain masks and 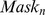 is the *n*-th brain mask (1 for brain tissues and 0 for non-brain tissues). The probability map has a value of 1 for the majority parts of the brain, except the boundaries where the voxel memberships are ambiguous. This probability map is warped onto the target image for initial brain extraction (using the binarized probability map) and is further used to guide the surface deformation for skull-stripping refinement.

**Figure 3:**
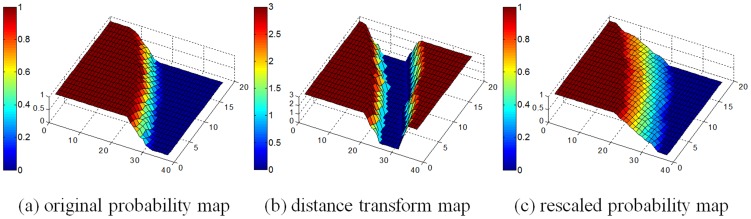
3D views of the rescaling process of the brain probability map.

**Figure 4:**
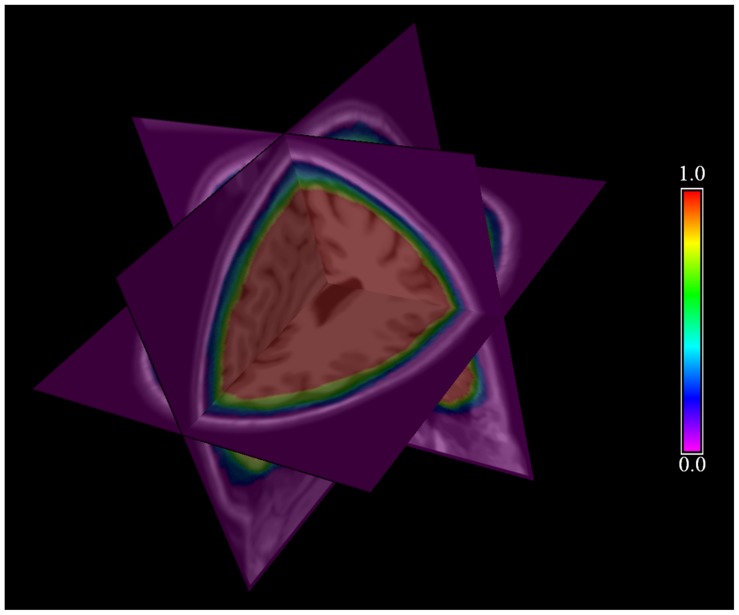
Axial, sagittal and coronal views of the brain probability map, overlaid on a brain MR image.

To account for inter-subject variation and potential estimation inaccuracy of the probability map due to the insufficient number of training images, the computed brain probability map is further rescaled based on a distance transform map to expand the ambiguous region (the region with the probability between 0 and 1). The value at each voxel in the distance transform map, which is calculated based on Danielsson's algorithm [43], indicates its distance to the nearest boundary voxel of the ambiguous region. Let 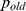 be the original probability calculated by Eq. (3), 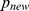 be the probability after rescaling, and 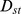 be the distance value. For each voxel 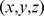, the new probability is calculated as:
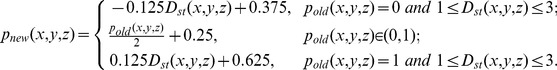
(4)Rescaling of the boundary is restricted to those voxels with distances to the boundary of the ambiguous region being no more than 3 voxels (determined experimentally). Specifically, the probability range of the original ambiguous ring region is rescaled to (0.25, 0.75) using the second term of Eq. (4). For regions with the original probability 0 (exterior to the ambiguous region), the probability is rescaled according to the first term of Eq. (4), with the rescaled probability of the region being [0, 0.25]. And for regions with the original probability 1 (interior to the ambiguous region), the probability of this region is rescaled to [0.75, 1] according to the third term of Eq. (4). Taking a small region of the probability map as an example (**Fig. 3**), **Fig. 3(a)** shows the original probability map; **Fig. 3(b)** shows the voxels within the distance of 3 voxels from the boundary of the ambiguous region in **Fig. 3(a)** (values greater than 3 voxels are truncated to 3 voxels); and **Fig. 3(c)** represents the new probability map after rescaling. An example of the final brain probability map is shown in **Fig. 4**. The probability map will be used to impose realistic shape and topological constraints for guiding the deformation of the surface as described in the following section, thus minimizing the chances of falling into less desirable sub-optimal regions.

Second, we introduce **the force guided by probability map**. Intensity information in images is commonly used to determine the boundary of the brain; however, intensity values are often influenced by artifacts introduced by noise or intensity inhomogeneity. Therefore, methods that rely on image intensity information alone are relatively susceptible to local minima. Our constructed population-specific probability map, which is adaptive to specific group, encapsulates prior information gathered from a population of real brain MR images. It is used to impose realistic shape and topological constraints for guiding the deformation of the surface, thus minimizing the chances of falling into less desirable sub-optimal regions. Therefore, the probability map guided force **F**_2_, derived from the warped brain probability5 map, is introduced as:
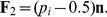
(5)Here 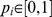 is the rescaled probability value of the current vertex 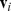. Similar to **F**_1_, **F**_2_ also acts in the direction of **n**. The force **F**_2_ accounts for the probability information learned from the training samples. When *p_i_* is larger than 0.5, indicating that the current point likely falls within the brain, an outward force is imposed; when *p_i_* is less than 0.5, indicating that the current point likely locates outside the brain, an inward force is imposed. The more the surface approaches the estimated boundary (*p_i_*≈0.5), the less the influential the force is.

### 3.2.3 Smoothness-constrained Force

**Figure 5:**
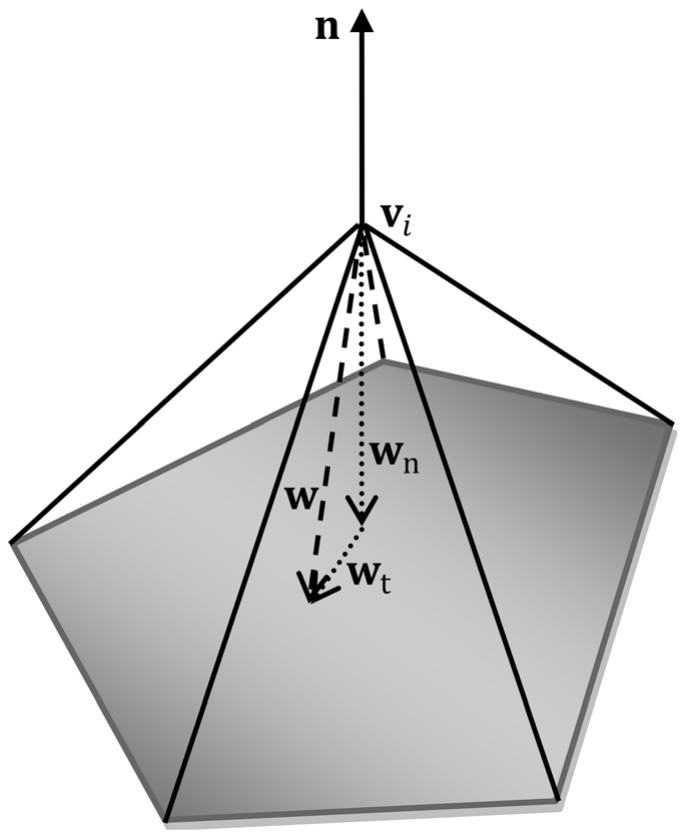
Local surface normal n and difference vector w of vertex v*_i_* with respect to its one-ring neighboring vertices.

The smoothness-constrained force is used to control the within-surface vertices equally spacing and smoothness during the surface evolvement, which tends to move the current vertex to line up with its neighbors and therefore increases the surface smoothness. First, the difference vector **w**(**Fig. 5**) is calculated between the position of the current vertex and the mean position of its one-ring neighboring vertices:
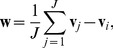
(6)with its normal and tangential components represented as 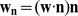 and 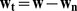, respectively. The difference vector **w** is used to take the current vertex to the mean position of the neighboring vertices in order to keep it aligned within the plane spanned by the neighboring points. Here *J* is the number of one-ring neighboring vertices for current vertex **v** *_i_*, and **v** *_j_* is the *j*-th neighboring vertex of **v** *_i_*.

**Figure 6:**
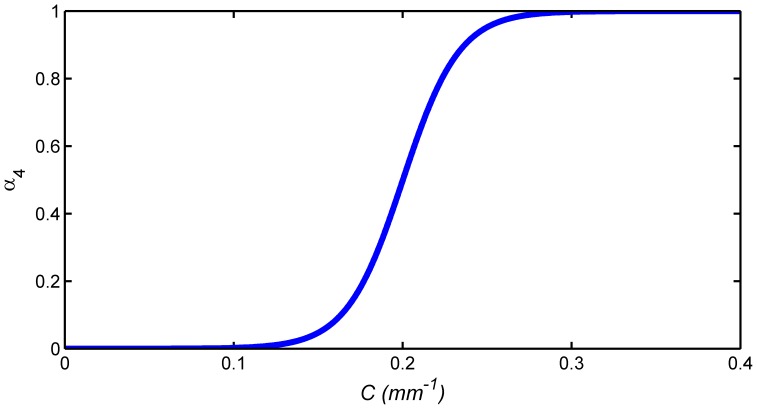
Smoothness update function with respect to the local brain surface curvature.

Thus the smoothness-constrained force is calculated as:
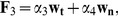
(7)Here the tangential and normal components of **w** are responsible for different roles. **w_t_** is tangential to the local surface, shifting the vertices along the surface to be equally spaced. **w_n_** acts parallel to the local surface normal **n**, moving the current vertex into the plane of its neighbors to increase the smoothness of the surface. In Eq. (7), *α*_3_ is set as 0.5 in this work; *α*_4_ is called the fractional update term and is adjusted adaptively. To ensure that the surface is sufficiently smooth and meanwhile avoid underestimation of the surface curvature, *α*_4_ is defined as a nonlinear function adaptive to the local surface geometry. This results in a curvature-reducing force that ensures smoothness of the surface during the evolution process. To achieve this, the local (absolute) curvature of the brain is determined first as 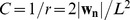. Here *r* is the local radius of the brain, and *L* is the mean inter-vertex distance calculated from each vertex to its one-ring neighboring vertices over the whole surface. *α*_4_ is defined as 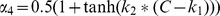, where 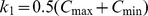 and 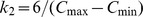 control the offset and scale of the sigmoid function, respectively (**Fig. 6**). The minimum and maximum curvature values 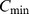 and 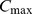 are empirically optimized based on typical geometries in the human brain and are respectively set to 0.1 mm^−1^ and 0.3 mm^−1^, corresponding to the local maximum and minimum radii of the brain 10 mm and 3.33 mm, respectively. The sigmoid function is used to penalize the high local mean surface curvature to achieve surface smoothness. Regions with low local mean surface curvature are not significantly affected by the curvature-reducing force. By updating of all the vertices on the surface, the ultimate surface is expected to be smooth with all vertices on the surface equally spaced.

In summary, at iteration *t*, for each vertex *i*, its update position is:
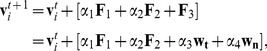
(8)where 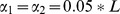, and *L* is the mean inter-vertex distance. During the surface evolution, the position of each vertex in the tessellated surface is updated with an estimated suitable position, which helps the surface gradually approach the target surface. The vertices reside in the real continuous space, instead of being constrained to the voxel grid locations. For searching an optimal solution, each individual movement is deliberately small, and surface updating is completed in many iterations (typically 1,000).

## Experimental Results

### 4.1 Compared Methods and Parameter Selection

Seven popularly used methods were evaluated in comparison with the proposed method: 1) BET [23], 2) Two-pass BET (2pBET), 3) BET-B: BET with bias field correction and neck cleanup (with option “-B”), 4) BSE [18], 5) HWA [25], 6) ROBEX [33], 7) AFNI [44]. For each of these methods (except ROBEX with no parameter tuning required and the proposed method), we determined for each image the best parameter setting by a parameter grid search. The grid search ranges for the parameters are shown in **Table 2**, which were determined according to those commonly used in the literature [3], [27], [33]. The actual ranges of the selected optimal values determined by grid search for each parameter of each method across all datasets are shown in the last two columns for “Humans” and “Non-human primates” respectively in **Table 2**.

**Table 2:** Grid search range and the optimal parameter range.

Method	Options	Default	Grid search range/status	Increment	Optimal parameter range	
					Humans	Non-human primates
**BET**	-f (fractional intensity threshold)	0.5	0.1∼0.8	0.05	0.25∼0.8	0.6∼0.8
	-g (vertical gradient)	0	−0.3∼0.2	0.1	−0.3∼0.1	−0.3∼0
**BET-B**	-f (fractional intensity threshold)	0.5	0.1∼0.8	0.05	0.15∼0.6	0.35∼0.8
	-g (vertical gradient)	0	−0.3∼0.2	0.1	0	0
	-B (bias field & neck cleanup)	OFF	ON		ON	ON
**BSE**	-d (diffusion constant)	25	5∼60	5	5∼40	5∼20
	-s (edge detection constant)	0.62	0.3∼0.8 (0.62 also investigated)	0.05	0.6∼0.75	0.65∼0.7
	-n (diffusion iterations)	3	3∼5	1	3∼5	3∼5
	-p (dilate final mask)	OFF	ON		ON	ON
**HWA**	default					
	-less (shrink the surface)					
	-more (expand the surface)					
	-atlas (use basic atlas information to correct the result)					
	-less -atlas					
	-more -atlas					
**AFNI**	-shrink_fac	0.6	0.3∼0.8 (suggested value 0.72 included)	0.05	0.3∼0.8	0.4∼0.7
	-shrink_fac_bot_lim	0.65	0.3∼0.8	0.05	0.3∼0.8	0.35∼0.75

#### BET, Two-pass BET (2pBET) and BET-B

BET in FSL (Version 4.1.6; http://www.fmrib.ox.ac.uk/fsl/) was used in this evaluation. For BET, parameters of the fractional intensity threshold “-*f*” and the vertical gradient “-*g*” were investigated. For two-pass BET, parameters of the fractional intensity threshold “-*f*” and the vertical gradient “-*g*” of the 1^st^ pass (values of which are denoted as 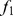 and 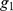) and the “-*f*” and “-*g*” of the 2^nd^ pass (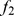 and 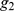) were all investigated. Ideally, these four parameters should be optimized simultaneously; however, to achieve this, one needs to search a parameters space with a few thousands of parameter combinations for each image, which is computationally intractable. Two-pass BET is executed in the following manner to reduce the computing cost: first, we obtain the best result by a parameter grid search of “-*f*” and “-*g*” (see **Table 2**); then a further grid search of “-*f*” and “-*g*” is executed based on the best result from the first step. For the following mutually exclusive options in BET: “-*B*” (bias field and neck cleanup), “-*S*” (eye and optic nerve cleanup) and “-*R*” (robust brain center estimation), based on our test with 20 randomly chosen HC subjects of the ADNI dataset, we found that “-*B*” consistently gives the better result, which is in agreement with the conclusion in Leung et al. and Shattuck et al. [3], [27]. We denote BET with the option “-*B*” turned on as “BET-B”. The ranges of the values for option “-*f*” and option “-*g*” are set to be the same as BET.

#### BSE

BSE in BrainSuite (Version 2009; http://www.loni.ucla.edu/Software/BrainSuite/) was used in this evaluation. The options “-*d*” (diffusion constant), “-*s*” (edge detection constant) and “-*n*” (diffusion iterations) were investigated. Our previous experience with BSE indicates that it has a tendency to erroneously exclude some brain tissues. As pointed out by Shattuck (the developer of BSE) et al. [3], the option “-*p*”, which dilates the final mask, is a new feature included in the latest version of BSE. Both Shattuck et al. [3] and Leung et al. [27] have found that this option gives improved results. Twenty subjects, similar to [27], were randomly selected from the HC group of ADNI dataset to be used to validate the choice of option “-*p*” (no figure shown). Our evaluation demonstrates consistently better skull-stripping results on these 20 subjects when the option “-*p*” was turned on, an observation that is similar to [3], [27].

#### HWA

HWA in FreeSurfer (Version 4.5.0; http://surfer.nmr.mgh.harvard.edu/) was used in this evaluation. In accordance with [3], [27], we investigated: “default”, “-less” (shrink the surface), “-more” (expand the surface), “-atlas” (use basic atlas information to correct the result), “-less -atlas” and “-more -atlas”.

#### ROBEX

ROBEX (Version 1.0; http://nmr.mgh.harvard.edu/~iglesias/ROBEX/flash.html#) was used as it is in this evaluation. No parameter tuning is required.

#### AFNI

AFNI (Version 2011-12-04; http://afni.nimh.nih.gov/afni/) was used in this evaluation. The parameters “-shrink_fac” (SF) and “-shrink_fac_bot_lim” (SFBL) were investigated. “-shrink_fac” is the parameter used to control the brain and non-brain intensity threshold, which is similar to the fractional intensity threshold in BET. Option “-shrink_fac_bot_lim” helps to reduce potential leakage below the cerebellum. Note that for the rhesus macaque dataset, one extra option “-monkey” was added.

### 4.2 Training Sample Size Selection

**Figure 7:**
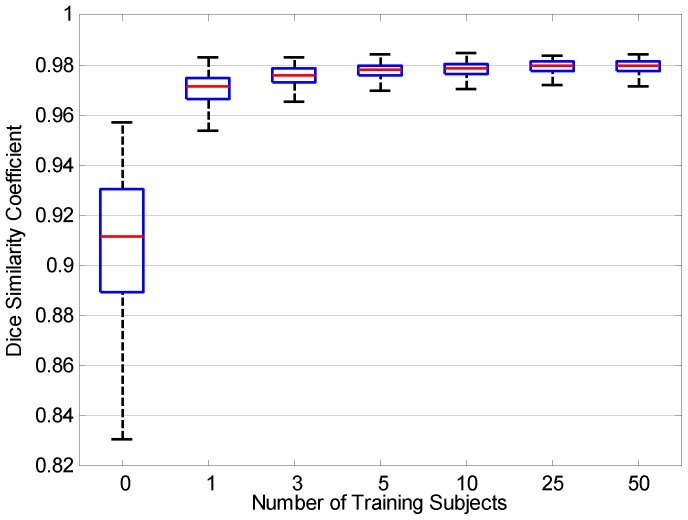
Dice coefficients given by different training sample sizes.

To find a suitable training sample size for the proposed method, we performed a series of experiments using the images of 230 subjects from the HC group in the ADNI dataset. The training sample sizes of 1, 3, 5, 10, 25, and 50 were evaluated. The 230 subjects were first randomly partitioned into two groups for training and testing. From the pool of training images, N (N = 1, 3, 5, 10, 25, and 50) images were randomly selected as the training data, and the trained results were then applied to all of the testing images for evaluation. For each training sample size N, random sub-sampling and the corresponding experiment was repeated 10 times. The Dice coefficient was used as the metric for evaluating our method with respect to expert-executed skull-stripping results. Let *A* and *B* represent the automatically extracted brain mask and the manually delineated brain mask, respectively. The Dice coefficient is defined as: 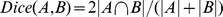. A fractional intensity threshold value (option “-*f*”) of 0.6 is used for all experiments. From **Fig. 7**, we can observe that the skull-stripping performance is significantly improved with the help of information gathered from the training data. For each box plot in the figure, the central mark is the median, the edges of the box are the 25^th^ and 75^th^ percentiles, and the whiskers extend to the most extreme data points without considering the outliers. Since the performance seems to flatten out from a sample size of 25 onwards, we chose 25 as the training sample size in subsequent experiments. The result from BET (not employing any training data, i.e., the number of training subjects = 0) is provided for comparison.

### 4.3 Evaluation of Parameter Sensitivity

**Figure 8:**
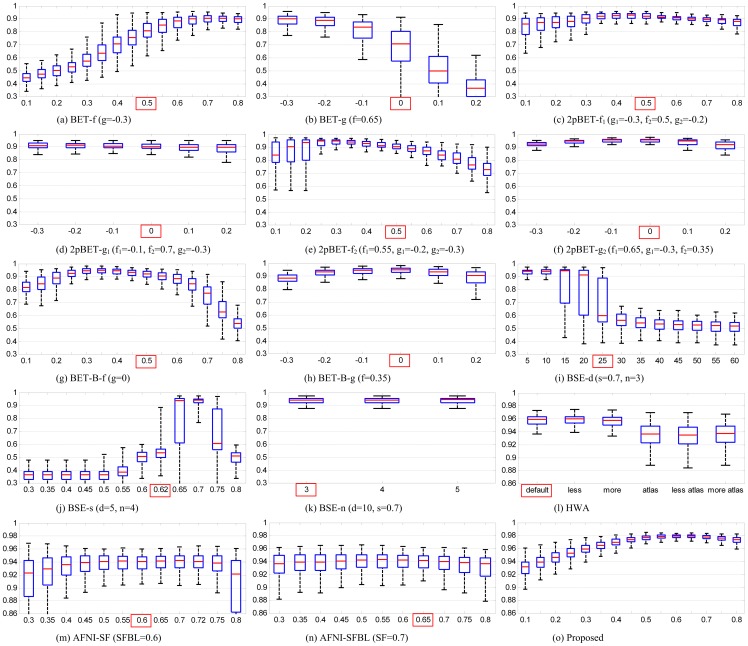
Comparison of parameter sensitivity for different methods: (a), (b) BET; (c), (d), (e), (f) 2pBET; (g), (h) BET-B; (i), (j), (k) BSE; (l) HWA; (m), (n) AFNI; (o) Proposed. The default parameter value of each option in the respective method is labeled using a red box. The vertical axes indicate the Dice coefficients. Note that for HWA, AFNI, and the proposed method, we used a different value range for the Dice coefficients for easier comparison.

To compare the parameter sensitivity of all methods in comparison, we evaluated the performance variation with respect to parameter changes. Dice coefficient was used as the performance evaluation metric. The HC group of the ADNI dataset was used for this evaluation. The best performance curve for each parameter with respect to all possible combinations of the other parameter(s) is determined (see **Fig. 8**). Taking BET for instance (options of interest: “-*f*” and “-*g*”), we changed the value of option “-*g*” from −0.3 to 0.2. The best performance curve of “-*f*” is found when parameter value of option “-*g*” is:
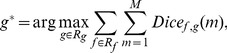
(9)where 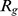 and 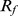 are the sets of parameters for options “-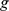
” and “-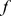
”; 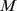 is the total number of testing subjects. We found that the best performance curve of “-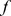
” occurred when the parameter for option “-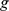
” was −0.3. With the same approach, the best performance curve for “-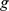
” was obtained when “-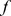
” was 0.65. It can be seen from **Fig. 8** that for most of the methods in comparison the default parameters are not necessarily the most effective parameters and that the ranges of optimal parameters are relatively narrow. We note that, as shown in **Fig. 8**(g) and **Fig. 8**(h), the vertical gradient “-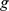
” does not affect the performance of BET-B significantly and the best performance for parameter “-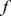
” is achieved with the default value of “-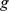
”. Therefore, “-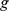
” is set to the default value (0) for the rest experiments of the BET-B method. There are two implementations for the BET-based methods: with or without option “-B”. Option “-B” is turned off in BET by default. As shown in **Fig. 8**(a), BET in its default setting achieves optimal performance at higher values of parameter “-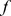
”, in line with the results in Popescu et al. [45]. For the case where option “-B” is turned on (denoted as “BET-B” in this paper), BET-B achieves optimal performance at lower values of parameter “-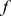
” as shown in **Fig. 8**(g), in agreement with the results in Leung et al. [27] and Popescu et al. [45]. BSE is sensitive to the changes of parameter values [33], especially parameter “-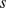
”. Small changes on the parameter value can result in large changes to the extracted brain result [33]. As shown in **Fig. 8**(j), BSE performs the best at 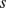 = 0.7, agreeing with the results in Shattuck et al. [3] and Leung et al. [27]. In contrast, our method is relatively parameter-insensitive (see **Fig. 8**(o)). When the fractional intensity threshold is within the range of 0.3∼0.8, the median Dice coefficients yielded by our method are consistently higher than 0.96.

### 4.4 Quantitative Evaluation on ADNI Dataset

#### Overlap Consistency

**Figure 9:**
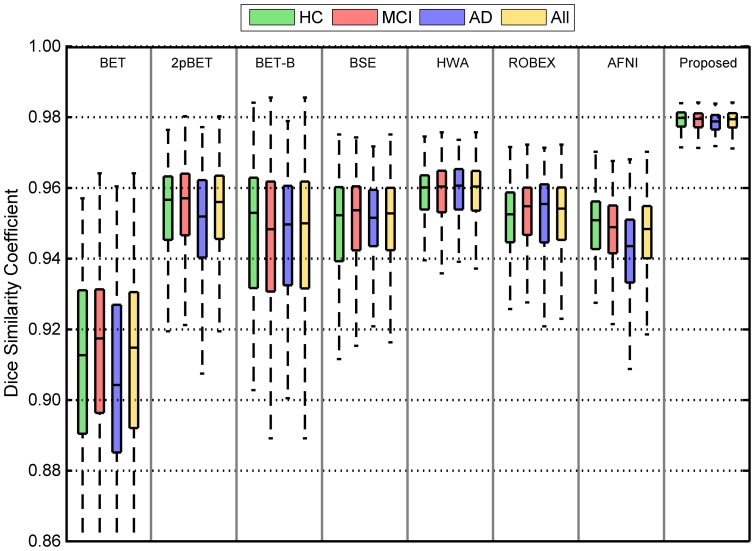
Distributions of Dice coefficients for the different methods and the different subject groups in the ADNI dataset.

**Figure 10:**
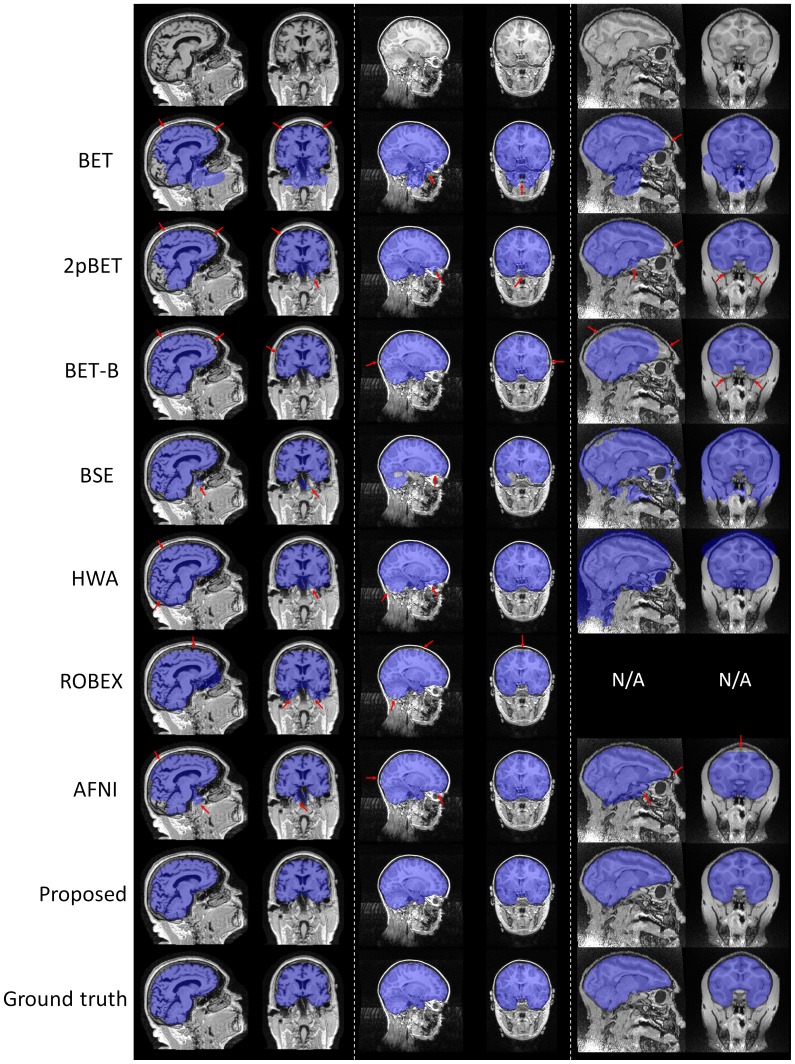
Typical brain extraction results for different methods. Left: adult; Middle: child; Right: rhesus macaque. Sagittal and coronal views are shown. Brain extraction results from different methods are highlighted in purple, and red arrows indicate some problematic regions.

Notably, we construct the probability map for each diagnostic group separately. As shown in **Fig. 9**, the proposed method yields consistently the best results for each group of the ADNI dataset when compared with all other methods, despite that only one single set of parameters was used for all images in our method. For each method compared (except ROBEX and the proposed method), the optimized set of parameters is obtained for each image, with the result providing the best Dice coefficient (based on its ground-truth brain mask) by grid search over a range of parameter values (see **Table 2**). Here a fractional intensity threshold value 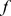 = 0.6 was used throughout the experiment for the proposed method. As can be seen, the proposed method does not show significant performance difference across different diagnosis groups, indicating that the proposed method is insensitive to different fractional intensity threshold values, which has also been confirmed in **Fig. 8**(o). A paired t-test (two-tailed test) is performed between each compared method and the proposed method based on the overall result, with the null hypothesis that the mean difference between the compared method and the proposed method is zero. We found that the differences between the proposed method and the compared methods are significant (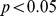). We can therefore conclude that the proposed method is significantly better than all other compared methods (BET, 2pBET, BET-B, BSE, HWA, ROBEX and AFNI). Representative results, compared with other methods, are provided in the first column of **Fig. 10** for the adult dataset. Some problematic regions are highlighted by red arrows. BET based methods typically over-skull-strip the posterior occipital cortex, anterior frontal cortex, while leave some non-brain tissue anterior to the brainstem and some dura in the superior parietal cortex unremoved. BSE leaves some part of the non-brain tissue unremoved. HWA keeps extra dura and non-brain tissue unremoved. ROBEX typically over-skull-strips the frontal cortex, the parietal cortex, and leaves some part of non-brain tissue unremoved (e.g., eyeballs and dura). AFNI typically over-skull-strips the posterior occipital cortex and part of the frontal cortex, while keeps some non-brain tissues anterior to the brainstem and some in the superior parietal cortex, which is similar with BET-based methods. The proposed method gives the best result by contrast.

#### False Positive (FP) and False Negative (FN)

**Figure 11:**
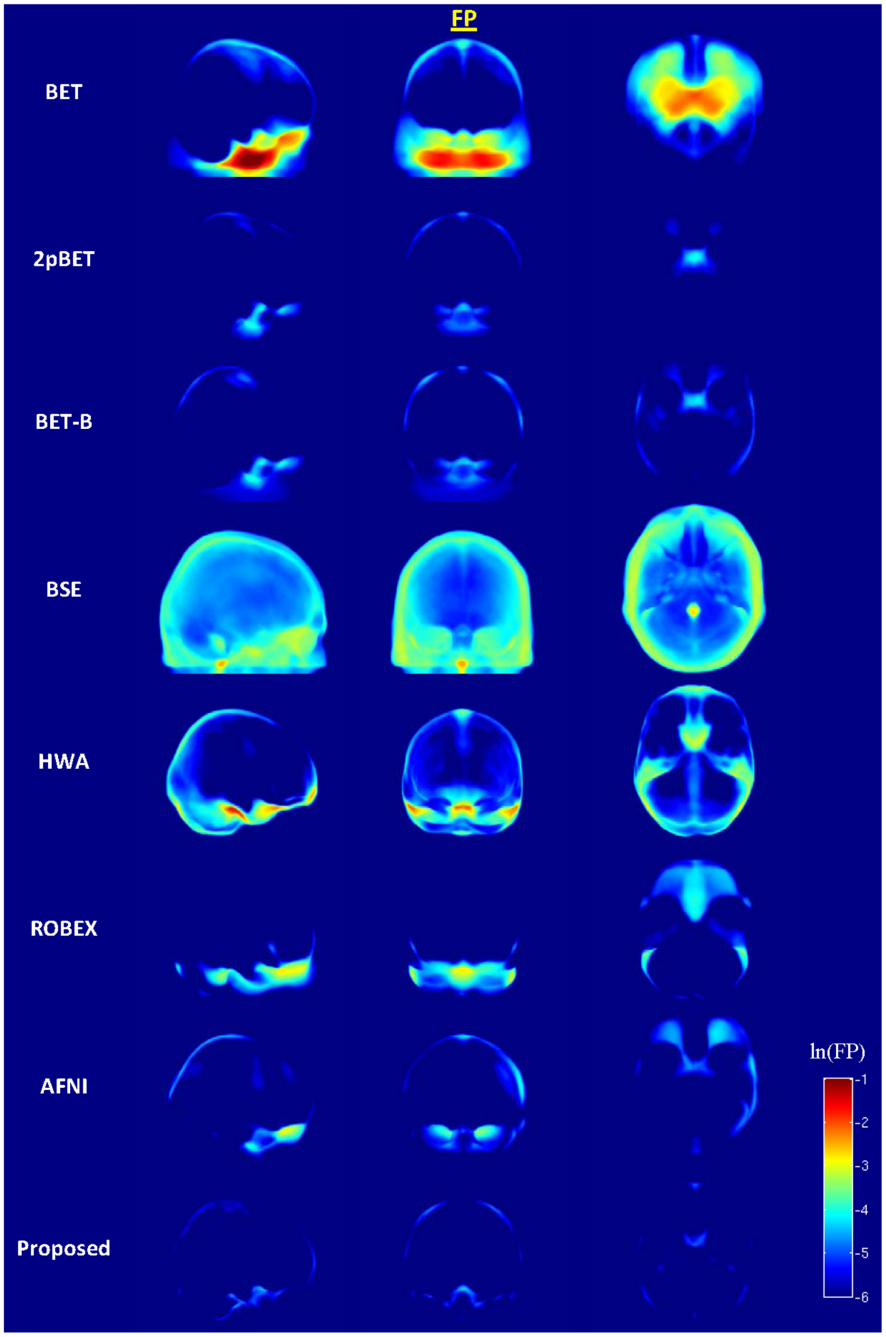
Sagittal, coronal, and axial views of the false-positive spatial probability maps for the different methods. Values are shown in natural logarithmic scale.

**Figure 12:**
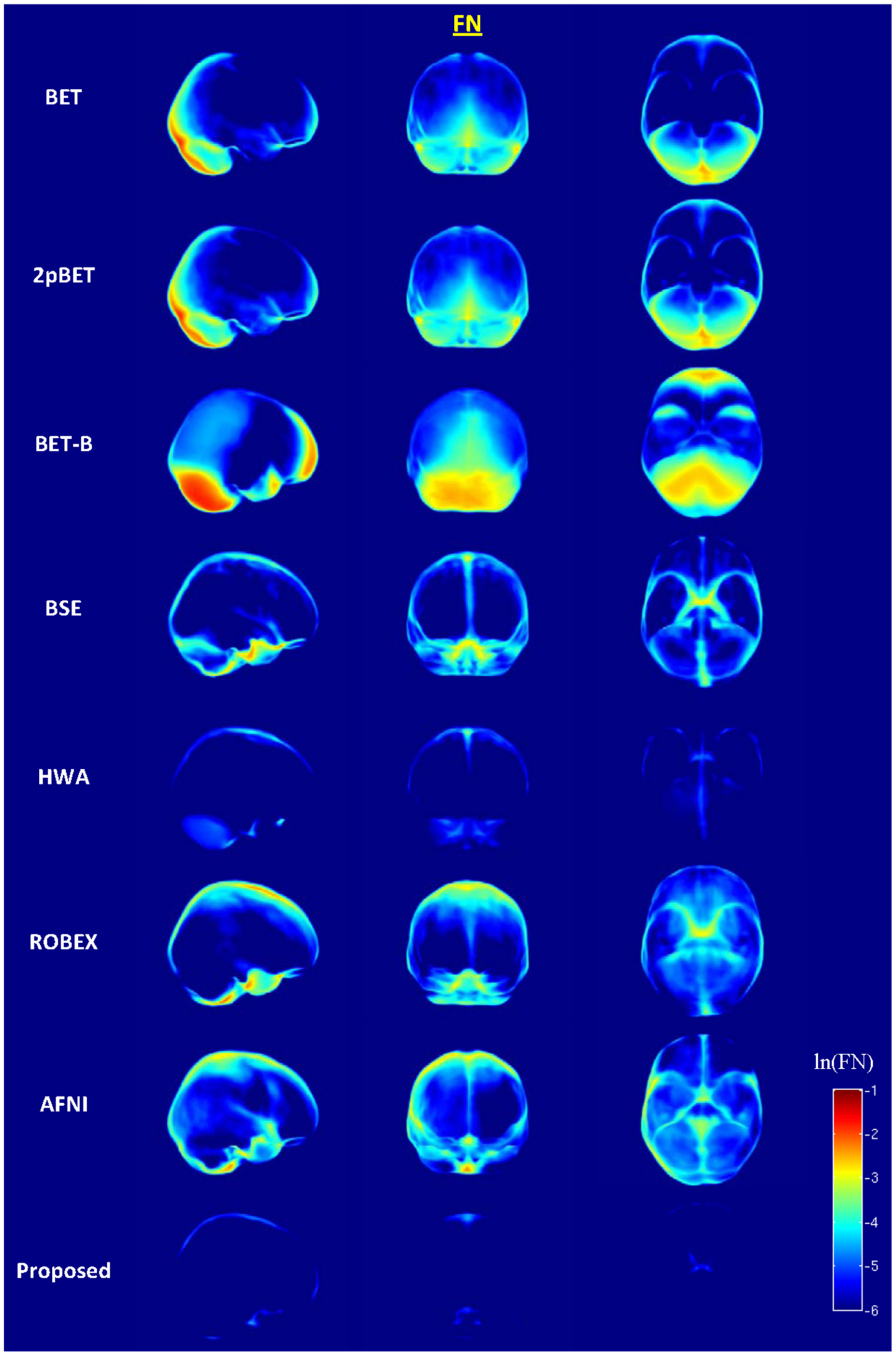
Sagittal, coronal, and axial views of the false-negative spatial probability maps for the different methods. Values are shown in natural logarithmic scale.

For closer inspection, **Fig. 11** and **Fig. 12** show the spatial distributions of false positives and false negatives given by the different methods for all diagnosis groups (HC, MCI and AD). If 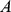 and 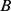 represent the automatically extracted brain mask and the manually delineated brain mask, respectively, false positive is defined as: 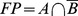 and false negative is defined as: 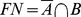. For each method, the average FP and FN images were obtained in the template space by concurrently considering all diagnosis groups. For visualization, we computed the 2D projection maps for axial, sagittal and coronal views by summing the values along the respective axis and dividing the outcome by the slice number along the axis. It can be observed from **Fig. 11** that BET tends to leave unremoved ventral tissue anterior to the brainstem. Two-pass BET (2pBET) works relatively well on excluding non-brain tissues, but leaves a significant amount of unremoved ventral tissue anterior to the brainstem, and some in the parietal lobes. BET-B works relatively well on excluding non-brain tissues, but leaves a significant amount of non-brain tissues in the ventral region, and some in the parietal and occipital lobes, similar to 2pBET. BSE fails to remove non-brain tissue from various regions of certain brains. HWA is relatively robust with little parameter tuning. However, it often under-segments the brain. As shown in **Fig. 11**, it leaves unremoved tissues along the ventral, frontal, occipital cortices and cerebellum regions. Note also that HWA usually fails to remove the dura matter, which is a documented shortcoming of the algorithm [33]. ROBEX tends to leave non-brain tissues in the ventral region. AFNI tends to leave unremoved ventral tissue anterior to the brainstem, and some in the parietal and occipital cortices. The proposed method performs the best in excluding non-brain tissues overall, despite the fact that there is a (negligibly) higher FPs in the frontal cortices compared with 2pBET and BET-B.

From **Fig. 12**, it can be observed that BET based methods typically over-skull-strip the anterior frontal cortices, anterior temporal cortices, superior parietal cortices, posterior occipital cortices, and the cerebellar areas. BSE over-skull-strips the border of the brain. HWA over-skull-strips the parietal and cerebellar regions. ROBEX typically tends to over-skull-strip the ventral tissue, frontal cortices, parietal cortices, and occipital cortices. As pointed out in [33], it tends to oversmooth the contour of the brain, and therefore tends to leave out some gray matter, which will present problems for cortical thickness estimation and gray matter volume measurement. AFNI typically tends to over-skull-strip the ventral tissue, frontal cortices, temporal cortices, parietal cortices, and occipital cortices, similar to ROBEX. The proposed method performs well on retaining brain tissues with just a little over-skull-stripping along the border of the parietal region. It has been observed that GM volume, GM density, and cortical thickness changes in the prefrontal and medial temporal areas are age-related [46], [47], [48]. Between children and adolescents, dramatic changes were observed in cortical thickness measurements in parietal cortices [49]. Accurate skull stripping of these regions is therefore especially important for developmental and aging research of the cerebral cortex. Overall, we can see that the proposed method works best in these regions.

#### Cumulative Histogram

**Figure 13:**
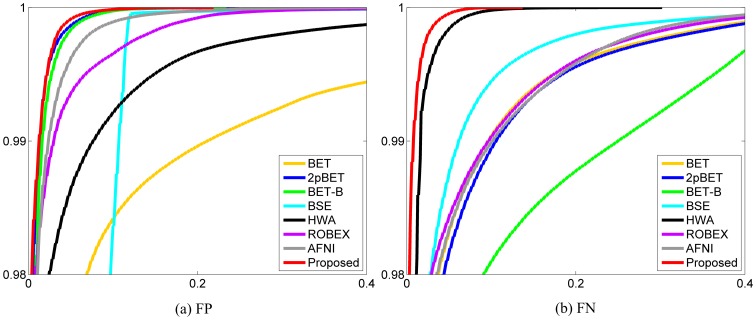
Cumulative histograms for FP and FN of different methods.

For better quantitative assessment, we computed the cumulative histograms of the average FP and FN images reported above. From **Fig. 13 (a)**, we can see that BET shows a higher fraction of FPs. HWA and BSE, while better than BET, also show a relative higher fraction of FPs. AFNI and ROBEX, while better than HWA, show a relative higher fraction of FPs than BET-B. BET-B, 2pBET and the proposed method show similar performance on FP, with the proposed method slightly superior. However, we can observe from **Fig. 13 (b)**, that BET-B shows a much higher fraction of FNs. BET, 2pBET, ROBEX, and AFNI, while better than BET-B, also show a relative higher fraction of FNs than BSE. HWA and the proposed method perform much better than the other methods on retaining brain tissues, with the proposed method being more superior.

#### Surface-to-Surface Distance

**Figure 14:**
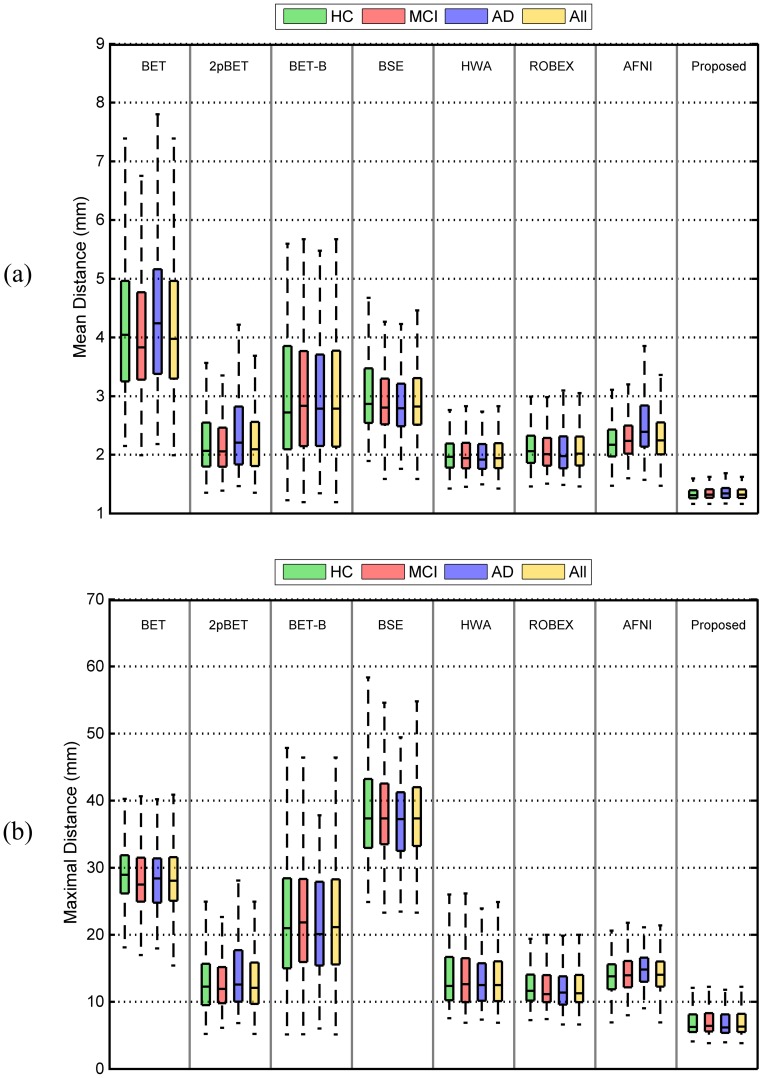
Comparison of different methods on the different subject groups of the ADNI dataset. Measurements include (a) mean symmetric and (b) maximal surface-to-surface distances.

We also computed the mean symmetric surface-to-surface distance and the maximal surface-to-surface distance as metrics for evaluating the skull-stripping accuracy, providing information on shape differences. The mean symmetric surface-to-surface distance, which measures from each voxel in the boundary of its estimated brain mask to the nearest boundary voxel in the ground truth and vice versa, provides a straightforward interpretation of the skull-stripping accuracy. **Fig. 14**(a) shows the statistics of the distributions of the mean boundary distances of all the testing data in the ADNI dataset, which is divided into three groups: HC, MCI and AD. The overall result is also provided. We found that the proposed method consistently gives the best result, with the mean symmetric surface-to-surface distances in overall only a little over 1 mm; while for the other methods, the mean symmetric surface-to-surface distances are at or above 2 mm.

Maximal surface-to-surface distance is also used to measure the degree of mismatch between the contours of a pair of brain masks. In contrast to the Dice coefficient, it penalizes cases in which two greatly overlapping objects have very different boundaries. Consistent with **Fig. 14**(a), **Fig. 14**(b) shows that the proposed method performs better than all other methods in comparison. Significant differences (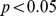) were found for both mean symmetric and maximal surface-to-surface distance. We therefore conclude that the proposed method is significantly better than all other compared methods (BET, 2pBET, BET-B, BSE, HWA, ROBEX and AFNI) for both metrics.

## 4.5 Quantitative Evaluation on OASIS Dataset

### Overlap Consistency

**Figure 15:**
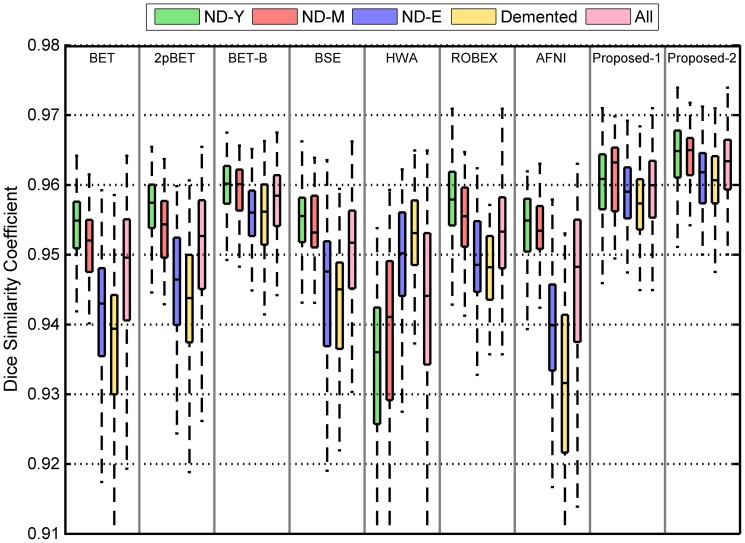
Distributions of Dice coefficients for the different methods and the different subject groups in the OASIS dataset. ND-Y, ND-M, and ND-E represent for young adults, middle-aged adults, and elderly adults in non-demented group, respectively.

We have both non-demented and demented groups for the OASIS dataset. To better account for differences in brain shapes across ages, we further divided the non-demented group into 3 age groups: young adults (ND-Y, age range: 18–39 years); middle-aged adults (ND-M, age range: 40–60 years) and elderly adults (ND-E, age range: 61–94 years). Similar to our evaluation based on the ADNI dataset, we compared, for each group dataset, the proposed method with BET, 2pBET, BET-B, BSE, HWA, ROBEX, and AFNI. For the proposed method, we used the ICBM high resolution template as the reference image to generate two brain probability maps (see **Fig. 15**): “Proposed-1” indicates the case where the probability map was generated from the HC group of the ADNI dataset in **Section 4.4**; “Proposed-2” indicates the case where the probability map was generated from the corresponding age group in the OASIS dataset (e.g. the probability map for the ND-Y is constructed by randomly selecting 25 training data from the same group). For each method compared, except our proposed method and ROBEX, we calculated for each image the best Dice coefficient given by the method by a grid search over a range of parameter values (see **Table 2**), as done previously. For the proposed method, a fractional intensity threshold value (option “-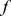
”) of 0.75 was used throughout the experiment. As shown in **Fig. 15**, Proposed-1, while giving good results, can be further improved by utilizing a probability map that is more specific to the population, i.e., Proposed-2. Note that BET, 2pBET, BSE, HWA, ROBEX and AFNI show significantly different performance in different age groups. In general, the performance of BET, 2pBET, BSE, ROBEX and AFNI decreases with the age; while that of HWA increases with the age. The median results of the proposed method Proposed-1 are better than any compared methods including BET-B in each of the diagnosis groups. Proposed-2 outperforms Proposed-1 and all other methods. Moreover, the proposed methods do not show significant differences in performance among different groups, demonstrating the robustness and consistency of the proposed framework. Overall, the proposed methods (Proposed-1 and Proposed-2) give significant improvements (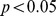) over all other compared methods (BET, 2pBET, BET-B, BSE, HWA, ROBEX and AFNI).

### Surface-to-surface Distance

**Figure 16:**
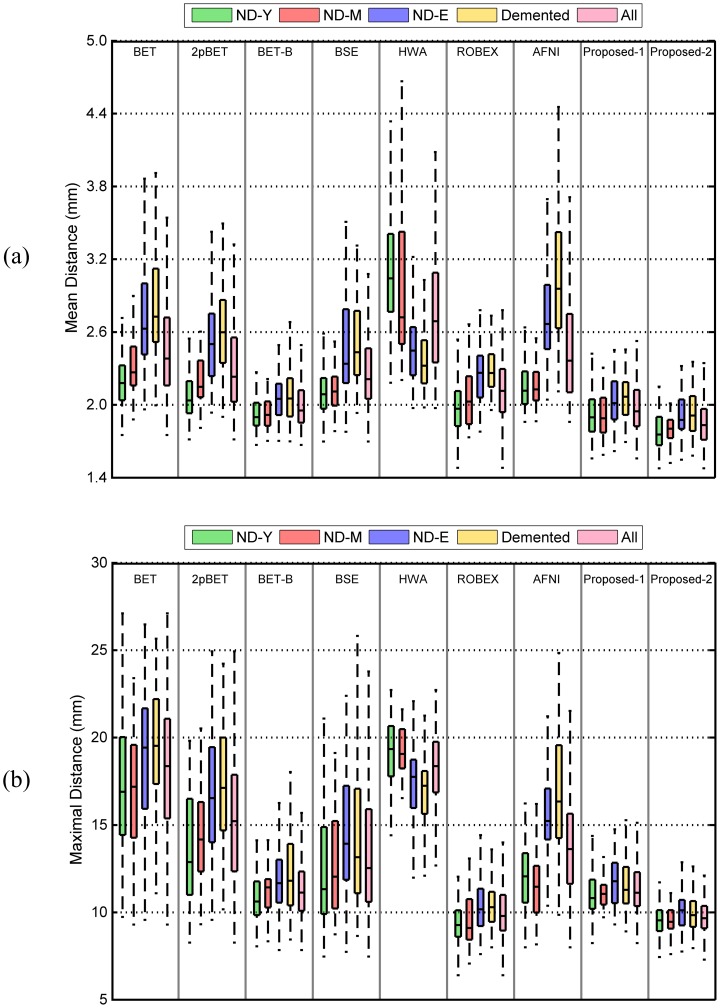
Comparison of different methods on the different subject groups of the OASIS dataset. Measurements include (a) mean symmetric and (b) maximal surface-to-surface distances.

Similar to the ADNI dataset, we evaluated the performance of the proposed method by measuring surface-to-surface distances in the OASIS dataset. The mean symmetric surface-to-surface distance results, as shown in **Fig. 16**(a), are consistent with the results of the experiment using the Dice coefficient for performance measurement. BET, 2pBET, BSE, HWA, ROBEX and AFNI show considerable different performance across age groups. In contrast, Proposed-1 and Proposed-2 consistently give the best results. Proposed-1 is comparable to BET-B even though a single set of parameters is used, and it is significantly superior to any other compared methods. Proposed-2 outperforms Proposed-1 and all compared methods. The maximal surface-to-surface distance results shown in **Fig. 16**(b) reflect that BET, 2pBET, BET-B, BSE, HWA and AFNI show significant performance differences across age groups. Similarly, Proposed-1 is comparable to BET-B though a single set of parameters is used. ROBEX is slightly better than Proposed-1, while Proposed-2 is slightly better than ROBEX. Among all methods, Proposed-2 consistently gives the smallest mean symmetric distances and maximal distances between the boundaries of the extracted brain masks and the ground truths. For mean symmetric surface-to-surface distance, Proposed-2 shows significant improvements (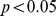) comparing with all other methods. There are also significant differences (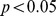) between Proposed-1 and all compared methods except BET-B. Similarly, for maximal surface-to-surface distance, the differences between the proposed methods and all compared methods are statistically significant (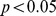). We therefore conclude that the Proposed-1 method is significantly better (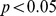) than the compared methods except BET-B and ROBEX; and the Proposed-2 method is significantly better (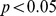) than all compared methods.

## 4.6 Quantitative Evaluation on NIHPD Dataset

**Figure 17:**
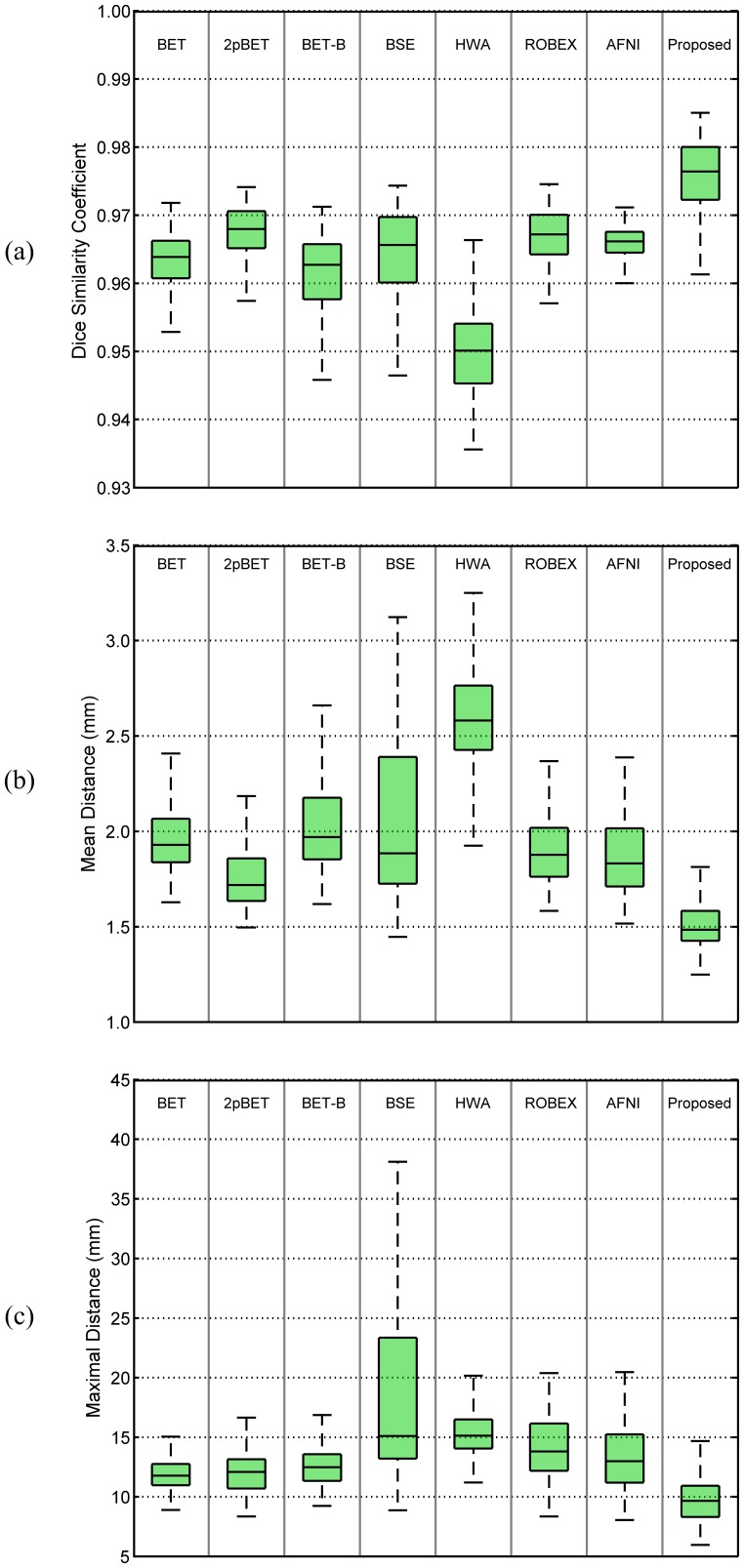
Comparison of different methods on the NIHPD dataset. Measurements include (a) Dice coefficients, (b) mean symmetric and (c) maximal surface-to-surface distances.

Complementary to the adult datasets used in previous evaluations, 150 subjects between 5 to 18 years of age were randomly selected from the NIHPD dataset, in which 25 subjects containing all age stages (5 to 18 years of age) were randomly selected from all subjects to construct the probability map for this age group. As can be observed from **Fig. 17**, the proposed method yields consistently the best results for both Dice coefficients and the surface-to-surface distances when compared with all other methods, despite the fact that only one single set of parameters (with option “-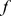
” be 0.75) was used for all images, whereas for all other methods (except ROBEX), the optimized parameters for each image was obtained by grid search. 2pBET outperforms BET, BET-B, BSE, HWA and AFNI. ROBEX has similar Dice coefficients with 2pBET. The proposed method has the best Dice coefficient and is significantly better (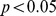) than all compared methods. 2pBET has relatively low mean surface-to-surface distance, which is a little superior to ROBEX and AFNI. While for maximal surface-to-surface distance, BET, BET-B and 2pBET have similar values, which perform better than ROBEX and AFNI. The proposed method achieves the best performance for both mean and maximal surface-to-surface distances, and is significantly superior (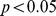) to all other methods in comparison.

The middle column in **Fig. 10** gives some typical results of different methods for the NIHPD Dataset, with some problematic regions being highlighted by red arrows. BET and 2pBET have some non-brain tissue unremoved inferior to the brain, including the eyeball. BET-B over-skull-strips the posterior occipital cortex, cerebellar region, superior temporal cortex and inferior frontal cortex. BSE has some brain tissue removed. HWA keeps a part of the eyeball unremoved, while over-skull-strips the cerebellar area. ROBEX over-skull-strips the frontal cortex, parietal cortex and cerebellar area, behaving similar with the adult result. AFNI over-skull-strips the posterior occipital cortex and parietal cortex, while keeping part of the eyeball unremoved. These problems are overcome using the proposed method.

## 4.7 Quantitative Evaluation on Rhesus Macaque Dataset

**Figure 18:**
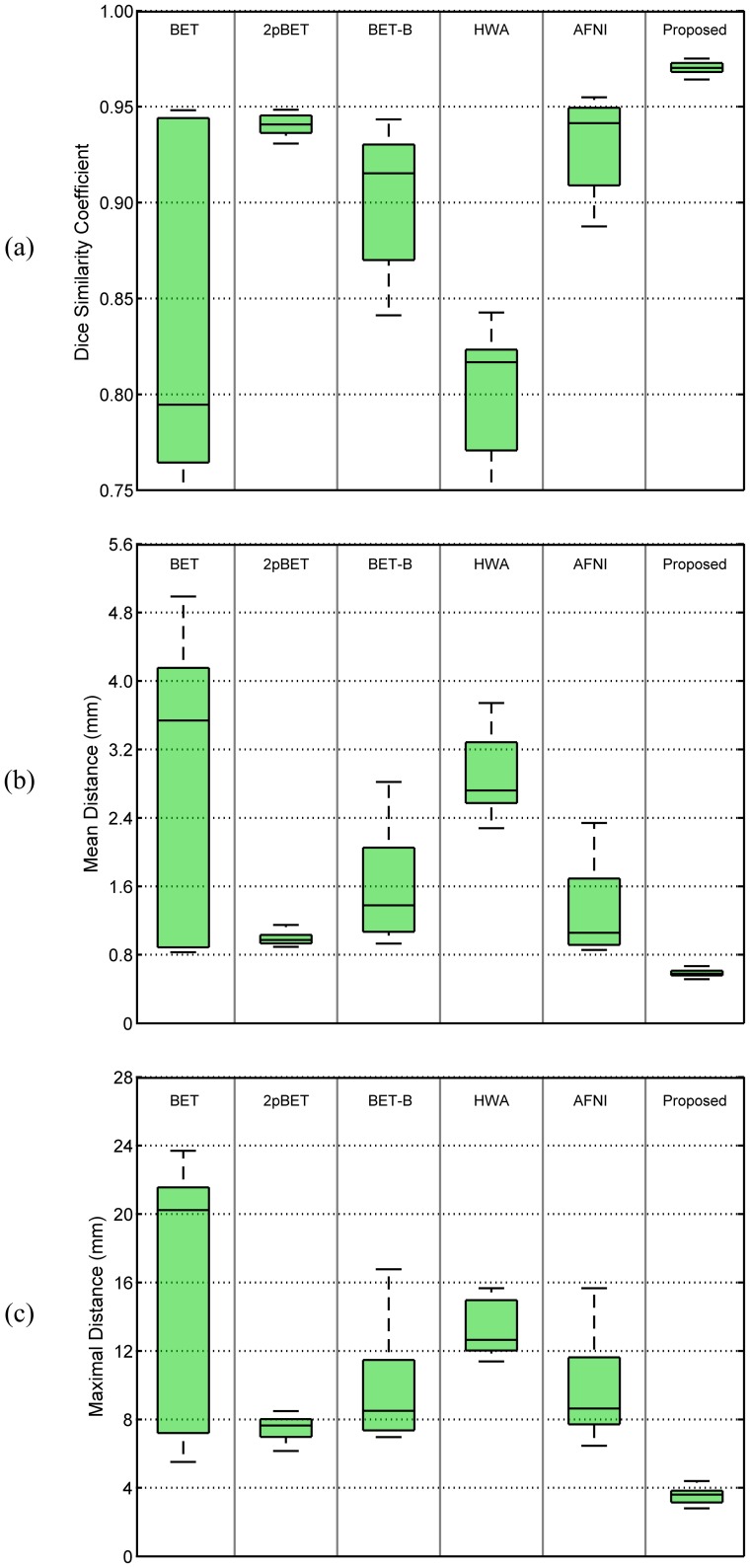
Comparison of different methods on the rhesus macaque dataset. Measurements include (a) Dice coefficients, (b) mean symmetric and (c) maximal surface-to-surface distances.

Because of the limit number of subjects in the rhesus macaque dataset compared to the human datasets, Leave-One-Out is employed here for validation of the proposed method. For each fold, one subject is used as the testing set, and the remaining 19 subjects are used as the training set to construct the probability map. This is repeated so that each subject in the dataset is used as the testing set once. For the proposed method, a fractional intensity threshold value of 0.85 was used throughout the experiment. For each method compared, as done in previous sections, the best Dice coefficient for each image given by each compared method is calculated by a grid search over a range of parameter values (see **Table 2**). Note that for the rhesus macaque dataset, AFNI has one extra option: “-monkey”. We found that 2pBET achieves better result compared with BET and BET-B methods (**Fig. 18**). Because of the utilization of monkey atlas information, AFNI achieves larger Dice coefficient on some subjects compared with 2pBET, yet the variation is also larger. The mean value of BSE is less than 0.75, and it is thus not shown in the figure. ROBEX does not work on the rhesus macaque data. One reason may be that, due to the large difference between human and rhesus macaque brain, ROBEX fails to perform the registration correctly using Elastix [50]. Our method gives the best result, with an overall Dice coefficient around 97%. Thus the proposed method is significantly superior (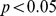) over all other compared methods. Significant differences (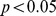) can also be found between the proposed method and the compared methods on both mean symmetric and maximal surface-to-surface distances. All methods give consistent performance on both mean symmetric and maximal surface-to-surface distance. BET-B and AFNI give the similar results for both mean symmetric and maximal surface-to-surface distances; while 2pBET performs better than BET-B and AFNI. The proposed method achieves the best result compared with all other methods.

Some typical results are given in the last column of **Fig. 10**, with some problematic regions highlighted by red arrows. BET based methods typically over-skull-strip the anterior frontal cortex and inferior temporal cortex, while leaving some non-brain tissue anterior to the brainstem unremoved. BSE and HWA do not work very well on the rhesus macaque dataset, and ROBEX fails to work on the rhesus macaque dataset as reported previously. AFNI typically over-skull-strips the frontal cortex and parietal cortex, while keeping some non-brain tissues anterior to the brainstem. Overall, the proposed method achieves the best result compared with other methods.

## 4.8 Computational Time

All programs run in Linux environment using a single thread on a 2.8GHz AMD Opteron Processor. The proposed method took about 2 minutes for registration using FLIRT and Demons, and less than 1 additional minute for skull-stripping an image. BET, BSE, and HWA typically took approximately 1 minute. BET-B took about 13 minutes, ROBEX took about 6 minutes, and AFNI took about 3 minutes. The computational time of our method is comparable to the existing methods.

## Conclusion

In this paper, an effective population-specific prior-knowledge guided framework was proposed for accurate and robust skull stripping on a wide variety of brain MR images consistently with the minimal parameter adjustment. We first performed an initial skull stripping by co-registration of an atlas, followed by a localized refinement phase under the guidance of population-specific prior information in a surface deformation scheme. Extensive evaluations were performed on diverse large-scale neuroimaging studies involving a significant number of brain MR images, e.g., the ADNI dataset, OASIS dataset, NIH pediatrics dataset, and rhesus macaque dataset. Experimental results on datasets across almost the entire human life span as well as nonhuman primates indicate that our proposed method outperforms the other compared methods such as BET, Two-pass BET, BET-B, BSE, HWA, ROBEX and AFNI, with only a single set of parameters, whereas the optimized set of parameters is obtained for each image in each compared method (except ROBEX) by a grid search. The software package of the proposed method has been released as part of Adult Brain Extraction and Analysis Toolbox (aBEAT) with graphical user interface, which is publicly available at http://www.nitrc.org/projects/abeat. The command version of the software package for various operating systems will be released in the future, which will be available at http://www.med.unc.edu/bric/ideagroup/free-softwares.
